# On the Use of the Muriate of Morphine in Toothache, Etc., Etc.

**Published:** 1847-10

**Authors:** 


19. *On the Use of Muriate of Morphine in Toothache, Frontal Neuralgia, and Neuralgia of the Fifth Pair of Nerves.*—M. Ebrard has always found toothache yield, in from half an hour to two hours, after friction of the gum on the affected side with muriate of morphine in powder. The first friction should be performed in the evening, at least three hours after the last meal, unless the severity of the pain prohibits delay. The patient should take a quarter of a grain of the salt on one of the fingers, previously moistened; rub it gently on the gum for about three minutes, then incline the head towards the affected side, avoid spitting or swallowing the saliva. so as to favor the contact of the salt with the affected part, and maintain this position for at least ten minntes. This process should be repeated in two hours if relief is not obtained. Should the pain return the day following, the application should be repeated. Half or three-quarters of a grain of the salt may be employed if necessary. The friction should not be repeated if headache, disposition to sleep, etc., occur.—*N. Y. Jour. of Med.*

